# Mechanical Behavior of Polymeric Materials: Recent Studies

**DOI:** 10.3390/polym16192821

**Published:** 2024-10-05

**Authors:** Emilia P. Collar, Jesús-María García-Martínez

**Affiliations:** Polymer Engineering Group (GIP), Polymer Science and Technology Institute (ICTP), Spanish National Research Council (CSIC), C/Juan de la Cierva, 3, 28006 Madrid, Spain

This Special Issue is devoted to one of the most exciting fields in polymer science and technology: the many factors that influence the properties of polymer-based materials. Therefore, it is pertinent to provide readers with some fundamental information on what is meant by failure in a polymer-based material (and thus in its mechanical properties). From a fundamental approach, fracture in a given material means overpassing the bond forces that keep its constituent atoms together. However, the ways in which these atoms are bonded as molecules and appear as very different types of supramolecular aggregates lead to complex questions, implying a heterogeneous character in these materials. In determining material strength capabilities, it has been undisputed since early studies by Griffith [1] that a fracture will always take place in the weakest region of a material; however, the search for empirical and semi-empirical approaches based on the results of conveniently designed mechanical testing procedures restricted to material or part performance still represent an open research question.

These considerations have become especially relevant in the case of heterogeneous materials based entirely or partially on organic polymers. From early times in materials research [2,3,4], it has been well established that the stress/strain relationship is not linear, because it is strongly dependent on time and temperature effects on the applied external force level and morphological changes in the material bulk as a result of environmental conditions [5,6,7]. This underscores the ongoing importance of research in this field fulfilling key research requirements and ensuring reproducible and reliable results. The development of inter-laboratory protocols and international standardized procedures is testament to the reliability of our results, and these efforts are crucial in providing robust mechanical parameter values [8,9,10,11,12,13]. These values should be suitable for incorporation into solid databases to feed polymeric part and material design software [14] in order to forecast the mechanical behavior of a polymeric material once it reaches the solid state after the processing steps that determine the emerging morphologies of the organic fractions in this type of material [15,16].

Ultimately, findings have made it clear that the study of mechanical energy interacts with matter and materials in different ways in different environments and conditions. This, together with the correct analysis of material responses, will continue to be vital in the development of mathematical models to assist in material design optimization within a general framework of sustainability, with the end purpose of avoiding catastrophic failure situations. A significant number of manuscripts were submitted for consideration for this Special Issue, but only a limited number were published following the rigorous revision process of *Polymers*. This Special Issue includes eleven exciting works related to this hotspot in polymer R&D. The articles compiled in this volume fully align with the philosophies mentioned above. The aim of this Editorial is not to elaborate on each of the texts but to encourage the reader to browse them in depth.

Sun et al. [17] present a finite element numerical simulation calculation for pavement structure load responses, considering that there is still a lack of research on polyurethane (PU) mixture composite pavement load responses because of our lack of understanding of the mechanical characteristics of PU mixture composite pavement. To fill this gap, this article analyses the mechanical properties of PU mixtures using the dynamic modulus test, the uniaxial penetration test, fatigue tests, and the finite element theory calculation method, to perform a load response calculation for an orthogonal-design composite pavement structure. The results show that PU mixtures exhibit more obvious elastic characteristics with good shear resistance, fatigue, and temperature stability and can be employed as shear and anti-fatigue layers. Using these data, a comparison with conventional pavement is performed and fascinating conclusions are drawn.

The article by Shishkovsky et al. [18] presents a study on the mechanical and thermomechanical performance of shape memory PLA parts by employing many sets, considering five variable printing parameters, in an FDM printing method. The results show that the temperature of the extruder and the nozzle diameter were the most significant parameters when it came to achieving better mechanical properties in the final part. In other words, this comprehensive study demonstrated a complex operational relationship between the mechanical and thermomechanical properties determined, combining the characteristics of a thermoplastic material with the shape memory effect and FDM printing parameters.

Vozniak et al. [19] investigated the mass transfer process of binary esters of acetic acid in poly(ethylene terephthalate) (PET), poly(ethylene terephthalate) with a high degree of glycol modification (PETG), and glycol-modified poly(cyclohexanedimethylene terephthalate) (PCTG), finding that the desorption rate of the complex ether at the equilibrium point was significantly lower than the sorption rate, depending on the type of polyester and the temperature and permitting the accumulation of ester in the polyester bulk. This effect is beneficial as a physical blowing agent in the filament extrusion additive manufacturing (AM) process, and it represents the main advantage of the obtention of nonbrittle foams compared to conventional polyester foams.

The work by Ehrmann et al. [20] is focused on the use of textile fabrics in fabricating stab-resistant garments with the advantage of being lightweight, considering most materials on the market. These research attempts are currently focused on textile fabrics, mostly through impregnation with shear-thickening fluids (STFs) or ceramic coatings, as well as on lightweight composites. In this work, the authors discuss different measurement methods, including dynamic and quasistatic methods, and the correlations of stab resistance with other physical properties jointly with an overview of recent developments and research on stab-resistant polymers, by employing different materials and combinations of materials and structures.

The investigation by Wildemann et al. [21] is focused on the effect of various types of external loadings on composite structures, leading to a decrease in mechanical properties in a system as a whole. For this purpose, many experimental investigations into the mechanical behavior of composites under uniaxial cyclic loading had previously been carried out. In contrast, in this study, new data on reducing composite materials’ mechanical characteristics under multiaxial cyclic loading were examined. Hence, these authors performed an experimental investigation into the mechanical behavior of fiberglass tubes under proportional cyclic loading and static and fatigue tests conducted under tension with torsion conditions. Further, the accumulation of structural damage was examined. Finally, the authors conclude that multiaxial cyclic loading significantly reduces the mechanical properties and must be considered in composite structure design.

The research article by Sebaey, Wagih et al. [22] demonstrates an enhancement in notch sensitivity in a hybrid carbon/epoxy (CFRP) composite with a Kevlar core sandwich compared to monotonic CFRP and Kevlar composites, bearing in mind that so-called fiber-reinforced plastic composites are sensitive to holes inducing out-of-plane stresses. The study is performed through open-hole tension (OHT) tests and further comparison of the open-hole tensile strength and strain and damage propagation. The results show that hybrid laminate has a lower notch sensitivity than CFRP and KFRP laminates because the strength reduction rate with the hole size is lower.

Belaadi et al. [23] present a comprehensive study of the elements that enhance the performance of biocomposites or sustainable ropes created from vegetable fibers, which need to have a high confidence interval (95% CI) for the mechanical characteristic data of performance materials in order to be useful for the design of parts due to the enormous variation in plant fibers properties. To achieve this, the authors implemented an experimental design involving four groups, varying the number of tests to determine the mechanical properties (modulus, strength, and strain at break) of sisal yarn in order to ensure that the CI was at least 95%.

The work by Peponi et al. [24] reports their study into the mechanical behavior of woven and non-woven PLA/OLA/MgO electrospun fibers in detail using the Box–Wilson surface response methodology as a follow-up to a previous work by the authors, where the diameters and thermal responses of these fibers were discussed in terms of the different amounts of magnesium oxide nanoparticles (MgO), as well as the oligomer (lactic acid, OLA) used as a plasticizer. The results suggest that these works can be strongly correlated. Critical points for both MgO and OLA were identified. Therefore, the approach presented by the authors permits the design of tailor-made electrospun nanocomposites with specific mechanical requirements.

The contribution by Koutsos et al. [25] addresses variability in the thermomechanical behavior of virgin and recycled polypropylene/high-density polyethylene blends without other components. The authors highlight the fact that understanding the performance variability in such blends is a crucial aspect in enabling recycled materials to re-enter the consumer market in terms of the circular economy, requiring further research to minimize the inhomogeneity of recycled materials. For these purposes, the authors performed a complete thermal and mechanical characterization of virgin and recycled polypropylene/high-density polyethylene blends, concluding that recycled and virgin blends are immiscible. This study was complemented by a dynamical mechanical analysis showing a slight variation in the storage modulus of recycled and virgin blends but lower alpha and beta relaxation temperatures in recycled blends. Additionally, the authors conclude that the tensile properties of recycled blends exhibit worse properties, which they explain.

The article authored by Cauich-Robriguez, Peponi et al. [26] explores the possibility of mimicking the architecture of human coronary artery native vessels for biomedical applications. The authors used roto-evaporation to engineer a three-layer polyurethane vascular graft (TVG). They synthesized two segmented polyurethanes using lysine (SPUUK) and ascorbic acid (SPUAA) to create the intima and adventitia layers, respectively. In contrast, the media layer was fabricated from a commercially available polyurethane and compared to single-layer vascular grafts (SVGs) from individual polyurethanes and a polyurethane blend (MVG). They found that the TVG exhibited the highest circumferential tensile strength and longitudinal forces compared to single-layer vascular grafts of lower thicknesses made from the same polyurethanes. The TVG also showed higher suture and burst strength values than native vessels. Additionally, the authors performed an indirect cytocompatibility test, showing 90 to 100% viability for all polyurethanes, surpassing the minimum 70% threshold needed for biomaterials deemed to have cytocompatibility. Except for SPUUK, all exhibited poor fibroblast adhesion and hemolysis values under the permissible limit of 5%, as well as longer coagulation times.

Finally, Minh et al. [27] present a study investigating the mechanical properties of coconut sawdust powder combined with polypropylene (PP) by observing the effect of the compatibilizer, wood powder (WP) content, and injection molding parameters on the properties of coconut wood powder composite (WPC). Based on a Taguchi experimental design, the authors performed an interesting investigation to determine the influential coordinates required to obtain optimized mechanical properties (tensile and flexural strength and hardness), aided by scanning electronic microscopy (SEM). They mainly concluded that the parameters influencing the ultimate properties follow a hierarchy, with the wood powder being the most influential and the melt temperature being the least influential.

To conclude, as the Guest Editors of this fascinating Special Issue, we can comfortably say that the topic “Mechanical Behavior of Polymeric Materials” represents an essential framework in the field of Polymer Science and Technology, now and in the near future. For this reason, a second Special Issue on this topic, to be published in 2025 in *Polymers*, is now in progress and open for submissions, and contributions are welcomed.
